# Molecular Detection of *Theileria* ovis and *Theleiria equi* in Livestock from Palestine

**DOI:** 10.1038/s41598-019-47965-0

**Published:** 2019-08-09

**Authors:** Kifaya Azmi, Amer Al-Jawabreh, Ziad Abdeen

**Affiliations:** 1 Biochemistry and Molecular Biology Department, Faculty of Medicine, Al-Quds University, Abu Deis, The West Bank Palestine; 2 Faculty of Medicine, Al-Quds Nutrition and Health Research Institute, Al-Quds University, Abu-Deis, P.O. Box 20760 The West Bank Palestine; 3 Al-Quds Public Health Society, Jerusalem, Palestine

**Keywords:** Diseases, Molecular biology

## Abstract

*Theileria* and *Babesia* are intracellular protozoan parasites infecting a wide range of animals. In Palestine, there is limited information on the prevalence of *Theileria* and *Babesia* spp. in livestock. We used PCR of the 18S ribosomal RNA gene followed by DNA sequencing to detect and identify parasite DNA in blood samples from sheep (n = 49), goats (n = 48), horses (n = 40), camels (n = 34), donkeys (n = 28) and mules (n = 2) from four districts of Palestine. DNA of *T*. *ovis* and *T*. *equi* was detected in 19 and 2 ovine blood samples, respectively. None of the camels, donkeys, and goats were positive for *T*. *ovis*. Sheep had a significantly higher rate of infection than other animals (P < 0.05). *Theileria ovis* is highly prevalent in sheep, while *T*. *equi* DNA was detected in a small proportion of the equids in Palestine.

## Introduction

Tick-borne haemoparasitic diseases caused by *Theileria*, *Babesia*, *Anaplasma*, and *Ehrlichia* are common in many regions of the world and result in a major burden on domestic animal production. Several pathogenic, moderately pathogenic, and non-pathogenic *Theileria* and *Babesia* species infect domestic ruminants. Ovine theileriosis; a major protozoal infection of sheep and goats^1^ is caused by several species of *Theileria*, of which, *Theileria lestoquardi* (syn. *Theileria hirci*) and *Theileria luwenshuni* (*Theileria* spp. China 1)^2^ are considered highly pathogenic. Other species such as *Theileria ovis* and *Theileria separata* cause subclinical infections in small ruminants^3,4^.

*Babesia* and *Theileria* species have been described in most livestock species and can cause significant economic losses to farmers. They are transmitted by a variety of ixodid ticks of the genera, *Ixodes*, *Rhipicephalus*, *Hyalomma*, *Amblyomma*, and *Haemaphysalis*^5^. However, due to a growing appreciation of the socio-economic importance of small ruminants, more attention is now being directed towards pathogens of sheep and goat. PCR analysis based on the 18S rRNA gene has been successfully applied to identify *Theileria* as well as *Babesia* species^6,7^. However, there is little information on infectious agents in livestock in Palestine, and the epidemiological aspects of theileriosis are not clearly understood, even though animal production is an important source of income in this country. So far, in a study done by Azmi and colleagues on ticks as hosts of pathogens in Palestine^8,9^, ovine species of *Theileria* were found in 5.4% of the ticks and were significantly associated with ticks from sheep and with the tick species *Rhipicephalus turanicus*. The aim of this study was to determine the prevalence of piroplasmid pathogens in domestic ruminants and equids from Palestine.

## Materials and Methods

### Animals and samples

Blood samples were taken from 201 healthy domestic animals including camels, horses, donkeys, sheep and goats between November 2015 and March 2016. The samples were collected from four localities in different parts of Palestine, representing the northern, southern, and eastern parts of the West Bank: Jenin, Nablus, Bethlehem and Jericho. Animals were located from 45 farms in Palestine. The geographic distribution of the animal population in Palestine was difficult to predict due to the limited knowledge about the farm distribution.

Blood was taken from the jugular vein and transported to the Al-Quds University where it was stored at −20 °C until DNA extraction. Blood collections were performed under the owners’ consent and the study was approved by the Internal Ethics Review Committee of the Al-Quds University. All experiments were performed in accordance with relevant guidelines and regulations.

### DNA Extraction, PCR amplification, and sequencing

DNA was extracted from 300 uL of blood using a commercial kit (Master Pure ^TM^ DNA purification kit for blood version II, Epicenter, Madison, WI, USA), following the manufacturer’s instructions as previously described^9^. The PCR reactions were performed using primers BJ1 (5′-GTC TTG TAA TTG GAA TGA TGG-3′) and BN2 (5′-TAG TTT ATG GTT AGG ACT ACG3′) which amplify a fragment of 460–540 bp of the 18S rRNA gene of piroplasmid infection including the genus *Babesia* and *Theileria* parasites as described previously^7,8^ and followed by sequencing to identify piroplasm DNA in positive samples.

All positive PCR products detectable by gel electrophoresis were sequenced at Hylabs in Jerusalem, Israel. The chromatograms were checked, and the sequences were assembled by the BioEdit software. The 18S rRNA sequences were trimmed and aligned using the Multalin Multiple sequence alignment tool (http://multalin.toulouse.inra.fr/multalin/). DNA sequences were compared with the GenBank database by the nucleotide sequence homology search facilitated by the National Centre for Biotechnology Information (NCBI) using the BLAST analysis database (http://blast.ncbi.nlm.nih.gov/Blast.cgi). The species’ identity of sequences was determined according to the closest BLAST match with an identity of 97–100% to GenBank accessions. All samples that were positive for piroplasmid*s* were confirmed by RFLP of the PCR product using the ApoI restriction enzyme as done by Azmi and colleagues^8^. To verify the source of the animal’s blood samples, mainly the horse and sheep blood, primers targeting the 12S and 16S mitochondrial rRNA gene, which amplify a polymorphic region from among a large diversity of species: 12-16SF (5′-ACACCGCCCGTCACCCTCC-3) and 12-16SR (5′-AACCAGCTATCACCAGGCTCG-3), were used^10^, and these samples were sequenced.

### Phylogenetic analysis

Phylogenetic analyses of the 18S rRNA sequences were performed by the Unweighted Pair Group Method with Arithmetic Mean (UPGMA) applying the neighbour joining and maximum likelihood algorithms. Phylogenetic tree analysis was conducted by the MEGA 6 program using the UPGMA module. The reliability of internal branches was assessed by bootstrapping with 1000 pseudoreplicates. Nodes with bootstrap support less than 70% were collapsed.

### Statistical analysis

Statistical analysis was carried out using the SPSS V.23.0 program. Pearson Chi-Square tests were used, and results were considered statistically significant if the p values were less than 0.05.

## Results

### Overall infection rates

A total of 201 domestic animals including 49 sheep (24.1%), 48 goats (23.6%), 40 horses (20.2%), 34 camels (16.7%), 28 donkeys (13.8%) and 2 mules (1.0%) from four localities in Palestine were included in the study. Samples were collected from 154 females and 48 male animals of all host species studied. The distribution of animals according to location is presented in Table 1.

**Table 1:** Infection with *Theileria* species in animals from different districts of Palestine.

Region (No.)	Number of animals in the study regions						Infection rate		
	Camel	Horse	Donkey	Mule	Sheep	Goat	PCR+ No. (%)	*T*. *ovis* No. (%)	*T*. *equi* No. (%)
Bethlehem (34)	2	2	9	1	10	10	6 (17.6)	5 (14.7)*	1 (2.9)*
Jenin (11)	11	0	0	0	0	0	0 (0.0)	0 (0.0)	0 (0.0)
Jericho (102)	21	35	18	1	17	10	5 (4.9)	4 (4.0)*,	0 (0.0)
Nablus (54)	0	3	1	0	22	28	11 (20.4)	10 (18.5)*	1 (1.9)*
Total (201)	34	40	28	2	49	48	21	19	2

Detection of *Theileria* DNA in animals from different districts of Palestine.

*sheep.

Based on PCR, followed by DNA sequencing, 22 domestic animals, i.e. 10.8% of the livestock was found to be positive for piroplasmid infection. *Theileria* spp. DNA was detected in the blood of 21 sheep (21/49; 42.9%), of which 19 were found positive for *T*. *ovis* and 2 were found positive for *T*. *equi*. None of the camels, donkeys, goats, and mules were positive for *T*. *ovis*. Successful identification of the horse blood was achieved using the 12S and 16S mitochondrial rRNA gene and DNA sequencing showed 100% identity with mitochondrial ribosomal operon (accession number: MG001413.1) from a Chinese horse. Sheep had a significantly higher rate of infection when compared with other studied animals (P < 0.05) (Table 1). Of the sheep studied, 43 were females (87.8%) and 6 were males (12.2%); of the 43 females, 15 were positive for *T*. *ovis* and 4 of the 6 males were positive for *T*. *ovis*. No significant association of PCR positivity was found with gender or location. No Babesia spp. were detected in any of the studied animals.

### Sequencing and phylogenetic analysis

**Figure 1:**
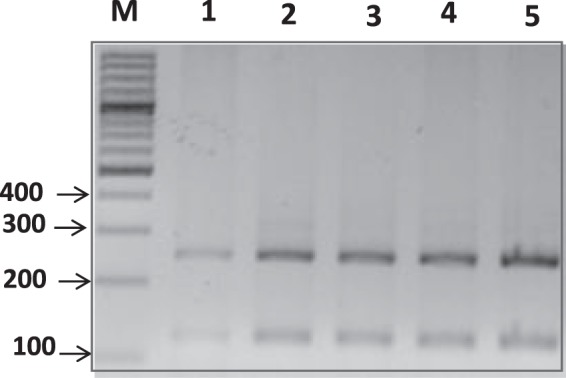
RFLP analysis of PCR products from representative DNA *T*. *ovis* samples of 18SrRNA gene following restriction with the ApoI restriction enzyme. Lane M, 100 bp DNA ladder; lanes 1–5: *T*. *ovis* from sheep.

All the PCR positive samples were sequenced. A neighbour joining phylogenetic analysis was used to reveal the relationship between the partially generated 18S rRNA gene sequence and other *Theileria* species’ sequences. The observed sequences were phylogenetically analysed to confirm their similarities. The *T*. *ovis* sequences described herein form a well-supported clade with all the other studied *T*. *ovis* sequences, whereas other *Theileria* sequences clearly clustered with *T*. *equi* sequences. The phylogenetic analysis for the 19 DNA sequences amplified in this study formed a well-supported clade which showed 100% identity with a *T*. *ovis* isolate from Turkey (Accession number: KU714608.1). PCR-RFLP patterns for all *T*. *ovis* amplicons showed the following patterns: 244, 121, 115, and 26 bp, which is characteristic of *T*. *ovis* (Fig. 1).

**Figure 2:**
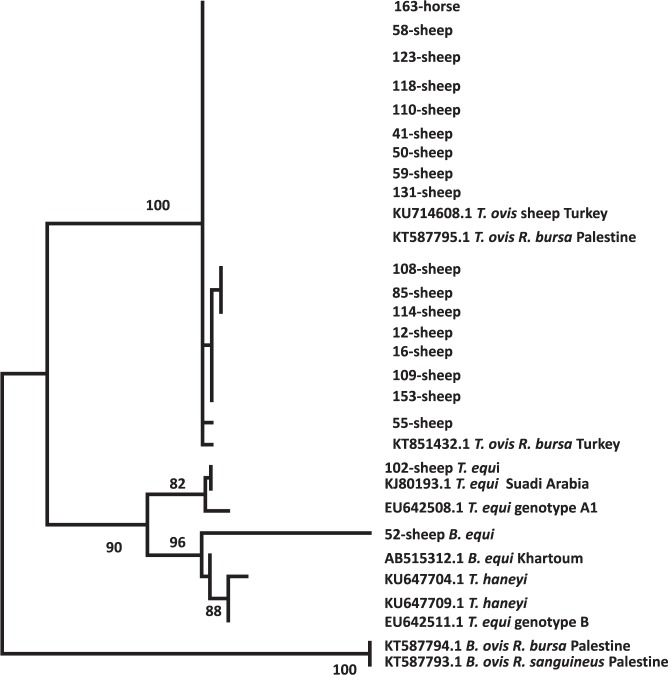
Neighbour joining phylogram comparing 489 bp 18S DNA *Theileria ovis* sequences to other sequences obtained from the GenBank database, constructed by the UPGMA method with bootstrap of 1000 replications using the MEGA software version 6.

**Figure 3:**
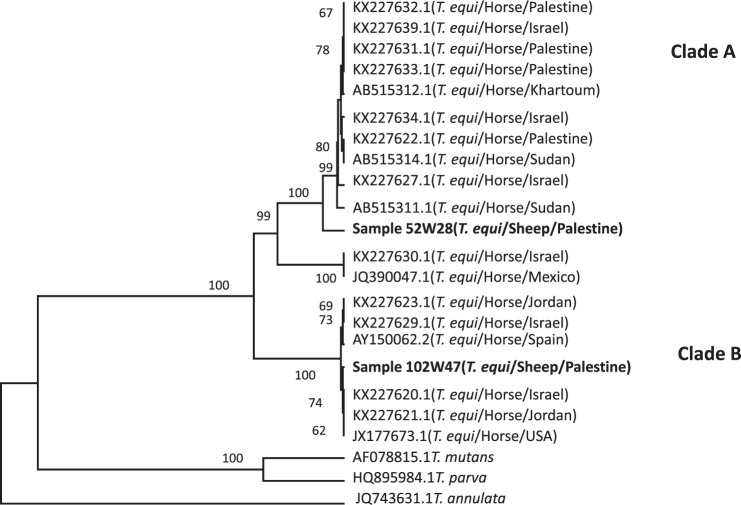
A maximum likelihood phylogram comparing 500 bp 18S DNA *Theileria equi* sequences from this study (in bold) to other sequences obtained from the GenBank database. Phylogenetic tree was constructed using the MEGA software version 6.

One of the two *T*. *equi* sequences obtained in this study showed 100% identity with *T*. *equi* (accession number: KJ801931.1) from a Saudi Arabian horse, while the second clustered with *T*. *equi* from a Sudanese horse (accession number: AB515312.1) (Fig. 2). Two sequences of the newly described *T*. *haneyi* (KU647704.1; Ku647709.1) clustered separately from *T*. *equi* and *T*. *ovis*^11^. The *T*. *equi* 18S rRNA gene of the two sequences fell into two genotypes, Clade A and Clade B (Fig. 3). Sample 52W28 grouped in Clade A, together with *T*. *equi* sequences from Palestine (KX227632, KX227631, KX227633.1, KX227622.1), Israel (KX227639.1, KX227634.1, KX227627.1) and Sudan (AB515311.1, AB515314.1). Also, within this clade, there was clustering of two sequences from Israel (KX227630) and Mexico (JQ390047.1) with a bootstrap value of 99. Sample 102W47 clustered together in Clade B with two sequences from Jordan (KX2276o23.1, KX227621), two from Israel (KX227629.1, KX227620), one from the USA (JX177673) and one from Spain (AY150062.2).

## Discussion

This is the first preliminary study in which molecular diagnostic techniques were used to screen for the presence of *Theileria* and *Babesia* spp. in livestock in Palestine. *Theileria ovis* was found to be the most prevalent species. A similar result with a high rate of *T*. *ovis* infection had been reported previously^6^ from ticks in Palestine (14.9% of the ticks from sheep), with *T*. *ovis* detected in *Rhipicephalus bursa*, *Rhipicephalus sanguineus* s.l. and *R*. *turanicus* that were collected while feeding on sheep. *T*. *ovis* is considered to be widely distributed in Asia, Europe and Africa^4^. The high prevalence of *T*. *ovis* in sheep (42.9%) in the present investigation was not surprising, since a high prevalence of this species was detected from sheep in Sudan (88.6%)^12^, Spain (18.9%)^13,14^ and Turkey (54.0 to 67.9%)^3,4^. Interestingly, *T*. *ovis* is considered as causing sub-clinical infection in small ruminants in contrast to the virulent *T*. *lestoquardi*^8,15,16^. No infection was detected in goats, whereas most of the sheep were infected. The higher frequency of infection in sheep compared to goats is in agreement with studies from other countries, such as Ethiopia, where *Theileria* spp. infection in sheep was also more common than in goats^17^ and also in a study from Turkey where 34.6% of the sheep and 10% of the goats surveyed were positive for *Theileria* spp.^18^. This variation in infection rates could be related to several factors such as the genetic variation among animals and the presence of tick species that act as vectors. No *Babesia ovis* infection was detected in the studied livestock. This suggests that *B*. *ovis* infection is rare in the surveyed area. This is in agreement with our previous findings^6^ that demonstrated the existence of a low level of *B*. *ovis* (0.6%) infection in *R*. *bursa* ticks collected from sheep in Palestine. *R*. *bursa* plays an important role as a vector of *B*. *ovis* and has been reported as the only vector for *B*. *ovis*^4^. The presence of *R*. *bursa* was relatively low among the ticks collected, 2.9%^6^, and almost all specimens of this tick were collected from sheep. This might explain why *B*. *ovis* was not as common as previously thought.

BLAST searches for DNA sequences of *T*. *ovis* from sheep, performed tor this study, indicated that they all clustered together. Comparison of the *T*. *ovis* 18S rRNA gene sequences obtained in this study indicates that *T*. *ovis* sequences are closely related to *T*. *ovis* sequences from Turkey (GenBank sequence: KU714608.1, KT851432.1) and from hard tick from Palestine (GenBank sequence: KT587795.1), with 100% query coverage. Furthermore, *T*. *ovis* from sheep grouped in different clades from *B*. *ovis* (GenBank sequence: KT587793.1 & KT587794.1) that were detected in ticks collected from our previous study^8^. In a previous study, *T*. *ovis* was significantly associated with ticks from sheep and with *R*. *turanicus* ticks^9^, and was the only species of *Theileria* found in ticks from Palestinian sheep. Interestingly, *T*. *equi* DNA was detected in the blood of two sheep. This is not surprising because our results agree with other studies in which *T*. *equi* DNA was identified in cattle, goats and sheep^19^ and it has also been found in dogs in Spain^20^. Equine piroplasmosis is caused by two intra erythrocytic protozoans, *T*. *equi* and *Babesia caballi*. *T*. *equi* is considered to cause a more virulent infection^21–26^. Our findings and other reports which indicate that *T*. *equi* also occurs in domestic ruminants further expand the host range of this organism^19,27^. *T*. *equi* is a major cause of disease in horses. In this study, none of the horses and donkeys were infected with *T*. *equi*. This may be because the collection was done in apparently healthy animals. The significance, extent, and consequences of infections with *T*. *equi* in domestic ruminants require further investigation.

Sequencing of a 18S rRNA PCR amplicon from a sheep in the current study was compatible with *T*. *equi* with 100% identity to a sequence from a horse from Saudi Arabia (KJ801931.1). Another sequence showed 95% identity with *T*. *equi* from a horse from Sudan (AB515312.1) and phylogenetically distinct from the novel species *T*. *haneyi* n. sp (KU647704.1 & KU647709.1), which is infective to equids, with an exceptional genomic diversity within the genus of *Theileria*. The two *T*. *haneyi* sequences (KU647704.1; Ku647709.1) were located in separate clusters of the dendogram^11^ data not shown. When comparing these sequences that were published from Saudi Arabian and Sudanese horses^28^ with sequences from Palestinian samples (KX227633, KX227631, KX227632) and from neighbouring countries such as Israel (Kx227639), there is a 96% identity by BLAST, and all these sequences cluster in clade A. In addition, all these sequences clustered with *T*. *equi* from Israel and Mexico (KX227630.1, JQ390047.1) with a high bootstrap value (99) as shown in Fig. 3. Although the small sample size may have affected these results, further molecular studies covering larger geographic areas targeting only *Theileria* and *Babesia* spp. are required to estimate the prevalence and economic importance of these infections in Palestine and to ascertain whether other piroplasmid species are present in the region. This preliminary survey is based on one molecular method only, which is reliable but cannot by itself indicate the presence of the actual parasite species, only of gene sequences similar to those of parasite species reported in previous studies. Accurate comparisons between the various regions of Palestine were not possible because the livestock animals sampled in each region were very different.

## Conclusion

This study demonstrated that ovine theileriosis is present in Palestine and suggested that *T*. *ovis* is the dominant piroplasmid agent in this region. Furthermore, evidence of *T*. *equi* infection in sheep is reported herein.
