# Author Correction: Molecular Detection of *Theileria* ovis and *Theleiria equi* in Livestock from Palestine

**DOI:** 10.1038/s41598-020-57959-y

**Published:** 2020-02-05

**Authors:** Kifaya Azmi, Amer Al-Jawabreh, Ziad Abdeen

**Affiliations:** 1 Biochemistry and Molecular Biology Department, Faculty of Medicine, Al-Quds University, Abu Deis, The West Bank Palestine; 2 Faculty of Medicine, Al-Quds Nutrition and Health Research Institute, Al-Quds University, Abu-Deis, P.O. Box 20760 The West Bank Palestine; 3 Al-Quds Public Health Society, Jerusalem, Palestine; 4 Department of Medical Laboratory Sciences, Faculty of Allied Health Sciences, Arab American University, Janin, Palestine

Correction to: *Scientific Reports* 10.1038/s41598-019-47965-0, published online 09 August 2019

The original version of this Article omitted an affiliation for Amer Al-Jawabreh. The correct affiliations for Amer Al-Jawabreh are listed below:

Al-Quds Public Health Society, Jerusalem, Palestine

Department of Medical Laboratory Sciences, Faculty of Allied Health Sciences, Arab American University, Janin, Palestine

This has now been corrected in the HTML and PDF versions of this Article.

